# The Economic Burden of Non-Typhoidal Salmonella and Invasive Non-Typhoidal *Salmonella* Infection: A Systematic Literature Review

**DOI:** 10.3390/vaccines12070758

**Published:** 2024-07-09

**Authors:** Sol Kim, Hyolim Kang, Jean-Louis Excler, Jerome H. Kim, Jung-Seok Lee

**Affiliations:** 1 International Vaccine Institute, Seoul 08826, Republic of Korea; sorukeem@gmail.com (S.K.); hyolim93@gmail.com (H.K.); jeanlouis.excler@ivi.int (J.-L.E.); jerome.kim@ivi.int (J.H.K.); 2 College of Natural Sciences, Seoul National University, Seoul 08826, Republic of Korea

**Keywords:** non-typhoidal *Salmonella*, invasive non-typhoidal *Salmonella*, economic burden, cost of illness

## Abstract

Non-typhoidal *Salmonella* (NTS) infection and invasive non-typhoidal *Salmonella* (iNTS) infection cause a significant global health and economic burden. This systematic review aims to investigate the reported economic burden of NTS and iNTS infection, identify research gaps, and suggest future research directions. Data from PubMed and Embase databases up to April 2022 were reviewed, and articles were screened based on predefined criteria. Cost data were extracted, categorized into direct medical costs (DMCs), direct non-medical costs (DNMCs), and indirect costs (ICs), and converted into US dollars (year 2022). Data primarily originated from high-income countries (37 out of 38), with limited representation from Africa and resource-limited settings. For inpatients, DMCs were the primary cost driver for both NTS and iNTS illnesses, with estimates ranging from USD 545.9 (Taiwan, a region of China) to USD 21,179.8 (Türkiye) for NTS and from USD 1973.1 (Taiwan, a region of China) to USD 32,507.5 (United States of America) for iNTS per case. DNMCs and ICs varied widely across studies. Although study quality improved over time, methodological differences persisted. This review underscores the lack of economic data on NTS and iNTS in resource-limited settings. It also highlights the need for economic burden data in resource-limited settings and a standardized approach to generate global datasets, which is critical for informing policy decisions, especially regarding future vaccines.

## 1. Introduction

Non-typhoidal *Salmonella* (NTS) infection, including invasive non-typhoidal *Salmonella* (invasive NTS, abbreviated iNTS), causes significant global morbidity and mortality, with a large economic burden impacting society [1,2]. While NTS infections mostly result in self-limited diarrheal enterocolitis with low case fatality, risk factors such as malnutrition, extremes of age (<5 years and ≥70 years), HIV, malaria, and sickle-cell disease increase susceptibility to iNTS infection [2,3]. iNTS disease occurs when the NTS organisms invade normally sterile sites, leading to sepsis, meningitis, pneumonia, arthritis, and osteomyelitis [2,4,5]. Studies have shown that approximately 6% of diarrheal NTS cases progress to bloodstream infections [6,7,8,9]. With a higher fatality rate than non-invasive infection, iNTS infection is a major cause of morbidity and mortality, especially in sub-Saharan Africa [2,10,11]. Majowicz et al. estimated 93.8 million cases of NTS gastroenteritis (95% UI 61.8–131.6), accounting for 155,000 (39,000–303,000) deaths worldwide in 2006 [12]. In 2010, the Global Burden of Disease (GBD) study estimated that NTS caused 4.84 million (3.82–5.94) DALYs [13] and 81,300 (61,800–101,700) deaths [14]. WHO estimates from the Foodborne Disease Burden Epidemiology Reference Group (FERG, 2007–2015) showed that non-typhoidal *Salmonella* enterica and invasive non-typhoidal *Salmonella* enterica were responsible for 4.38 million (3.24–7.18) and 3.9 million (2.4–5.8) DALYs, respectively [15]. Furthermore, foodborne DALYs caused by NTS and iNTS were the largest (4.07 million) among the 22 foodborne enteric diseases globally [15]. More recently, the GBD 2019 estimates report 594,000 (486,000–718,000) cases of iNTS disease, resulting in 79,000 (43,000–124,000) deaths and 6.11 million (3.32–9.71) DALYs globally [16]. In children under 5, NTS (36% deaths) and iNTS (45% deaths) had a greater impact than typhoid fever (17% deaths) and paratyphoid fever (2% deaths) [16]. Most iNTS cases occur in Sub-Saharan Africa, with incidence rates exceeding 100 cases per 100,000 person-year in population at risk [17]. The 2017 GBD report estimated a case-fatality rate of 14.5% (9.2–21.1), with higher rates in children, the elderly, and people living with HIV infection [2]. Other studies report even higher case fatality rates of 20% in Mali, Bangladesh, and Vietnam [17].

Various prevention strategies have been implemented to control NTS disease, targeting animal production, food industries, consumers, and national surveillance [18,19]. Despite these efforts, significant under-reporting persists due to barriers to healthcare access and insufficient attention from public health authorities at national and global levels [1,2,20,21,22]. Additionally, the lack of point-of-care biomarkers and rapid diagnostic tools, such as serological or PCR-based tests, make the clinical diagnosis of NTS and iNTS infection challenging, particularly in low-resource settings such as Africa, where blood culture facilities are limited [11,23]. The absence of an available vaccine against NTS infections exacerbates these challenges, contributing to a significant public health burden. 

No systematic attempt has been made to estimate the per-case cost incurred due to NTS and iNTS diseases [24,25]. This review aims to examine the existing studies on the economic burden of NTS and iNTS infections to date, identify gaps, and suggest future research needs.

## 2. Materials and Methods

A systematic literature review was conducted in accordance with the Preferred Reporting Items for Systematic Reviews and Meta-Analyses 2020 guidelines and its checklist (Supplementary Materials; Table S1) [26]. PubMed and Embase were searched using search strategies constructed with free-texts, MeSH-Terms, and Emtree terms, adapted for each database. The search included publications from inception to April 2022, with the last search conducted on 29 April 2022. Two categories of search terms were used: (1) disease category, including terms related to non-typhoidal Salmonella and invasive non-typhoidal Salmonella, and (2) economic burden category, including terms related to the cost of illness. All search terms within each of the two categories were combined using the “OR” operator, and the two categories were combined using the “AND” operator. No filters or limits were applied, and there were no language restrictions. A detailed list of search strategies is presented in Table 1. Duplicate articles were identified and removed using EndNote software. The initial screening of titles and abstracts was conducted independently by two reviewers (SK, HLK). Studies indicating the cost of NTS or iNTS were selected for a second screening. After the initial screening, the list of studies for full-text review was shared, and discrepancies were resolved through discussion. The two reviewers independently carried out the full-text review of 77 articles using pre-determined inclusion and exclusion criteria (Table 2). The reference lists of the selected studies were further reviewed by the two reviewers. Any inconsistencies between the reviewers were resolved with the assistance of a third researcher (JSL).

The quality of the selected articles was assessed using a quality assessment tool (Supplementary Materials; Table S2) adapted from the cost-of-illness evaluation checklist proposed by Larg and Moss (2011) [27]. The results of the quality assessment (high, medium, and low) are presented in Table S3.

Relevant data were extracted from each included study using a standardized template in a Microsoft Excel workbook. Data extraction was initially performed by SK and HLK and cross-checked for accuracy. The extracted variables included the title of the article, authors’ names, publication year, cost year (study period), study location, disease indication, serovars, currency of the cost, cost item and description, cost type, cost perspective, cost source, duration of illness (number of visits), and other cost-related information identified in the articles. Cost types were categorized into direct medical costs (DMCs), direct non-medical costs (DNMCs), and indirect costs (ICs). DMC refers to costs incurred by patient management such as hospital stays, physician consultations, laboratory tests, and medications. DMC was further classified into DMC inpatient (DMC IP) and DMC outpatient (DMC OP). When DMCs were not specific to costs by inpatient or outpatient healthcare services, they were categorized as DMC non-specific (DMC NS). DNMC includes costs not directly related to healthcare services, such as transportation, food, and accommodation. IC encompasses expenses borne by patients or their families due to work absenteeism related to the illness. Data costs related to outbreak control and the value of lost lives were extracted but not included in the review results. All cost components were calculated as per-case costs whenever possible, by dividing aggregated costs for a group of patients by the number of patients. All costs were converted to US dollars (USD) and adjusted to 2022 values using the official exchange rate and the GDP Deflator provided by the World Bank [28].

## 3. Results

**Figure 1:**
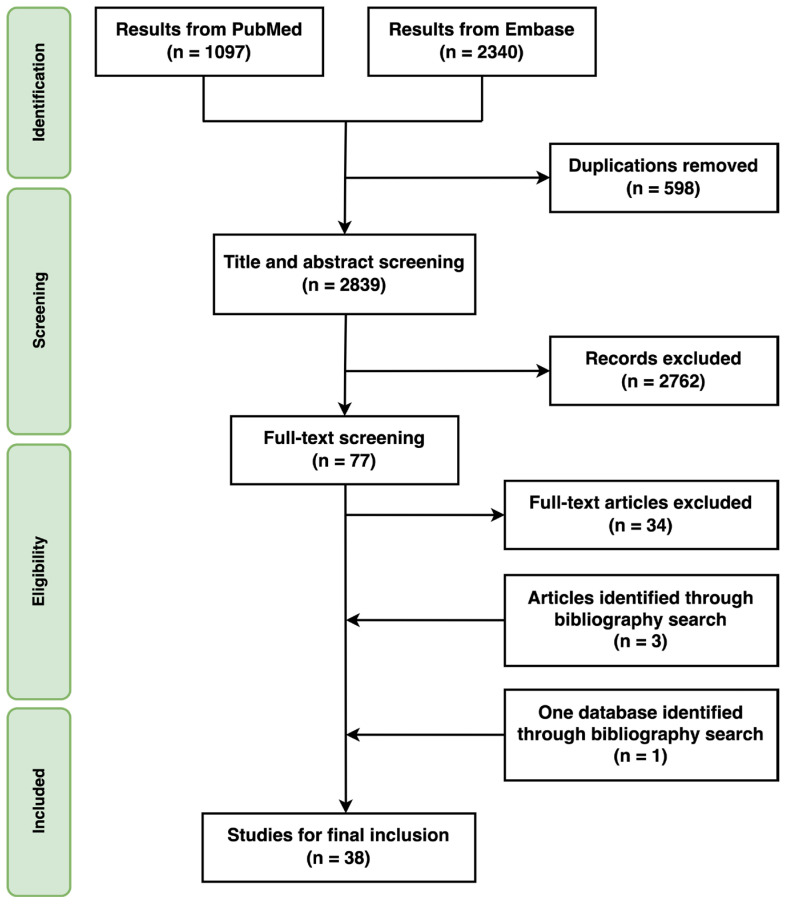
Study selection.

The literature search yielded 2839 articles after removing duplicates, of which 77 were identified for full-text review based on their titles and abstracts. Among these 77 articles, 34 met the inclusion and exclusion criteria. Additionally, three articles [29,30,31] and one database [32] were identified through bibliography searches and included in the final review (Figure 1). 

Among the 38 studies, 4 articles contained data related to the economic burden of iNTS [30,33,34,35]. Evidence for NTS disease came from 11 countries: Australia, Canada, Hong Kong SAR, China (hereinafter Hong Kong), the Netherlands, Poland, Spain, Sweden, Taiwan, a region of China (hereinafter Taiwan), Türkiye, the United Kingdom (UK), and the United States of America (US). Evidence for iNTS was found in three countries: Spain, Taiwan, and the US. The income level of each country, classified by the World Bank (2021), was compared to assess the distribution of data [36]. Only one study [37] was conducted in an upper middle-income country (Türkiye) in 2009, while all other studies were from high-income countries. There were no studies from lower middle-income and low-income countries for both NTS and iNTS diseases. Geographically, no studies were conducted in Africa, and only two studies were conducted in Asia (Taiwan [5,35] and Hong Kong [38]).

Publication years ranged from 1978 to 2021. All studies used economic evaluation methods from various perspectives, such as societal, the healthcare system, and the patient. More than half (n = 25, 66%) of the studies used a societal perspective, encompassing DMC, DNMC, and IC. Eight [5,35,37,39,40,41,42,43] out of thirty-eight studies used the healthcare system perspective, focusing on government and healthcare facility costs. Five studies used the patient perspective, which focused on the medical expenses incurred by the patient and their families, excluding indirect costs [29,33,34,38,44]. 

Among the 38 studies reviewed, 13 aimed to estimate the economic burden of NTS outbreaks [20,22,37,39,40,41,45,46,47,48,49,50,51], of which five were in hospital settings [37,39,40,41,47]. Todd et al. estimated the economic burden of multiple *Salmonella* community outbreaks in the UK, US, Canada, Sweden, and Australia across different years [20], and Scharff et al. did so in the US from 1994 to 2009, using national surveillance data [51]. None of the outbreak studies included iNTS cases. Economic evidence related to iNTS was derived from either hospital databases (US and Spain) or national claims databases (US and Taiwan) [30,33,34,35]. A summary of the extracted components from the included studies is provided in Table S3 in Supplementary Materials.

The reported DMC IP was the primary cost driver for the economic burden of NTS and iNTS illnesses, ranging from USD 545.9 (Taiwan) to USD 21,179.8 (Türkiye) for NTS illnesses and from USD 1973.1 (Taiwan) to USD 32,507.5 (US) for iNTS illnesses per case. Considering the significant inflation surge in Türkiye in 2022, around 72% [36], the second highest DMC IP for NTS illness was USD 19,788.7 from the US. The DMC OP reported was lower than the DMC IP, ranging from USD 17.4 (UK) to USD 739.8 (US) for NTS illnesses, with only one study from the US [30] estimating DMC OP for iNTS as USD 469.4 per case. The IC ranged from USD 188.9 in Australia to USD 3028.8 in the UK per case for NTS illnesses. The IC for iNTS illnesses, as reported by Adhikari et al. (2001) from the US, was USD 1768.8 [30]. DNMCs were assessed in 14 studies, ranging from USD 4.3 in the Netherlands to USD 18,261.2 in Türkiye [20,31,37,40,41,45,46,47,49,51,52,53,54,55]. Five studies (13%) did not report costs by components but aggregated total costs [19,39,50,56,57]. Costs reported from each study are presented in Table 3 (NTS illnesses) and Table 4 (iNTS illnesses). When costs could not be categorized into DMC, DNMC, and IC, total costs were presented as total costs (TC) in the tables (all costs are expressed in US dollars at 2022 value).

To better understand the economic burden relative to each country’s economic context, we calculated the healthcare costs as a percentage of the country’s GDP per capita [28]. For NTS illnesses, the DMCs IP as a percentage of GDP per capita ranged from 0.3% (UK) to 198.4% (Türkiye) (Table 3). This significant variation indicates the differing economic impacts on patients in different countries, with Türkiye experiencing a disproportionately high economic burden due to its lower GDP per capita and exceptionally high inflation rate in 2022 [36]. For iNTS illnesses, the DMCs IP as a percentage of GDP per capita ranged from 0.6% (US) to 42.6% (US) (Table 4). The high percentages for iNTS reflect the severe financial impact of invasive infections, particularly in high-income countries like the US, where healthcare costs are substantial. Countries with national healthcare systems, such as Taiwan and the UK, generally report lower out-of-pocket expenses for patients compared to those without such systems, impacting the overall economic burden experienced by individuals.

Regarding the study quality of the 37 articles, 20 studies were rated as high quality [5,22,30,35,37,38,42,43,44,49,50,53,54,55,57,58,60,61,62,63], 12 as medium quality [29,31,33,34,41,46,47,48,51,52,56,59], and 5 as low quality [19,20,39,40,45]. Study quality was assessed using a set of questions regarding the analytical framework, methodology and data, and analysis and reporting [27]. The quality assessment tool was not applicable to the one database established by the Economic Research Service (ERS) at the U.S. Department of Agriculture (USDA) due to its structure [32]. Detailed information is available in Supplementary Materials (Table S2). It is noteworthy that the quality of cost-of-illness studies has improved during a span of 44 years (1978–2021), particularly in analysis and reporting, despite methods still being varied among the recent studies. 

Data on the duration of illness were also collected, as shown in Table 5. Hayes et al. reported the number of outpatient visits for NTS illness management to be 1.6 per case [46]. No other study clearly indicated the number of outpatient visits related to NTS illness management, and none did so for iNTS illness. Hospital bed-days or length of hospitalization was reported to be 7.5 days (95% Confidence Interval 6.0–8.9) in 16 studies for NTS illness per case and 12.6 days (95% CI 8.6–16.5) in three studies for iNTS illness per case. Roberts and Sockett, Herrick et al., and Ailes et al. presented the duration of NTS illness to be 12, 2, and 4 days (median) per case, respectively, without specifying the type of healthcare resource (inpatient or outpatient) [22,49,56].

## 4. Discussion

At the global level, the economic burden of NTS and iNTS is predominantly informed by data from a limited number of high-income countries, mainly the US and the UK. Among the 37 articles and 1 database reviewed, 15 included data from the US and 10 from the UK. Notably, no economic evaluation was conducted in the African region, despite the high burden of NTS disease in sub-Saharan Africa [2,11,17,23]. Additionally, there was no evidence from any lower middle-income or low-income countries, highlighting the scarcity of cost data for NTS and iNTS diseases in resource-limited settings, as previously reported [13,14,23]. Direct comparisons of costs across studies are challenging due to the varied approaches and definitions of the costs used. Furthermore, several studies did not separately describe cost categories (DMC, DNMC, IC) and reported combined total costs instead [19,39,50,56,57]. 

This study has limitations. Although we employed a systematic approach to identifying literature relevant to the economic burden of NTS and iNTS diseases by searching multiple databases with different search terms, there remains a possibility of missing some literature. Costs associated with iNTS diseases may not have been separately estimated and reported if the study did not specifically aim to do so. Additionally, the diverse methods and outcome measures of cost-of-illness studies made it infeasible to conduct a meta-analysis. 

To understand the global economic burden of NTS and iNTS, a consensus on standardized methods of economic evaluation across nations is needed to generate reliable data. Efforts to improve diagnostics, understand disease sources, and study routes of transmission should be combined with high-quality primary data to assess the economic burden of NTS and iNTS. A comprehensive understanding of the economic burden is essential for making evidence-based policy decisions at national and global levels.

## 5. Conclusions

The findings of this review highlight the significant lack of economic evidence for NTS and iNTS in the regions most in need, particularly Africa. This disparity underscores the disproportionate attention and resources concentrated in high-income countries, especially within a limited number of nations. Currently, there are a few *Salmonella* combination vaccine candidates in early clinical trial phases, including bivalent NTS vaccines (GSK Vaccines Institute for Global Health (GVGH), Boston Children’s Hospital) and trivalent NTS and typhoid vaccines (University of Maryland and Bharat Biotechnology, GVGH, SK Bioscience and International Vaccine Institute) [64]. McLennan et al. emphasized that a vaccine-related strategy with multivalent *Salmonella* vaccines for control should be applicable in LMIC settings, considering the lack of diagnostic capacity and adequate treatment options [64]. To support the development of NTS and iNTS vaccines by informing investments and policy decisions, a better understanding of the economic burden of NTS and iNTS diseases, especially in endemic regions, is essential. Public health communities must make concerted efforts to identify and address the substantial burden of NTS and iNTS diseases globally.

## Figures and Tables

**Table 1:** Search strategies.

	PubMed	Embase
**Disease category**	invasive non typhoidal salmonell * ints nontyph * invasive AND non? typhoid invasive AND nts non? typhoid non? typhoid * non AND typhoid fever typhimurium enteritidis choleraesuis salmonella AND heidelberg salmonella AND dublin salmonella AND newport salmonella AND virchow salmonella AND concord salmonella AND brancaster salmonella AND infantis salmonella AND isangi salmonella AND freetown	salmonella enterica serovar heidelberg salmonella enterica serovar dublin salmonella enterica serovar choleraesuis salmonella enterica serovar virchow salmonella enterica serovar infantis salmonella enterica serovar typhimurium salmonella enterica serovar enteritis salmonella food poisoning non AND typhoid* AND salmonella invasive nontyphoidal salmonella disease ints invasive AND nts nontyph * non?typh * no * AND typh * nts non AND typhoid * typhimurium enteritidis choleraesuis salmonella AND newport salmonella AND concord salmonella AND brancaster salmonella AND isangi salmonella AND freetown
**Economic burden category**	costs and cost analysis cost of illness cost–benefit analysis cost allocation healthcare costs health expenditures hospital costs hospital charges cost cost * cost minimi * cost effective * economic burden economic burden *	cost cost benefit analysis cost effectiveness analysis hospital cost cost minimi * cost of illness cost* cost effective* economic burden

**Table 2:** Inclusion and exclusion criteria.

**Include if**	▪ The study includes the direct medical costs OR direct non-medical costs OR indirect costs of NTS-related treatment/healthcare source utilization. ▪ The study includes costs incurred due to NTS disease from primary data or secondary data. ▪ The study estimates the costs incurred due to NTS disease by modelling.
**Exclude if**	▪ The costs are related to animal disease or food control. ▪ The study includes costs incurred due to NTS with other diseases combined such as other foodborne diseases, typhoid, and paratyphoid (costs not specific to NTS). ▪ Full text is written in languages other than English.

**Table 3:** Healthcare costs by cost type related to non-typhoidal *Salmonella* diseases (USD in 2022).

No.	Author	Country	Cost Year	Original Currency	Cost Type	Cost in USD (2022)	Unit	% of GDP Per Capita ^§^
1	Cohen et al. [45]	US	1976	USD				
					DMC^r^	1785.2	per case	2.3
					DMC IP	5514.7	per case	7.2
					DMC OP	262.6	per case	0.3
					DNMC	408.0	per case	0.5
					IC	1143.3	per case	1.5
2	Curtin et al. [31]	Canada		CAD				
			1982		DMC^c^	753.8	per case	1.4
					DMC IP	3256.1	per case	5.9
					DMC OP	160.0	per case	0.3
			1978		DNMC	59.2	per case	0.1
			1978		IC	1842.3	per case	3.3
3	Todd et al. * [20]	Multiple countries	1983	USD				
		UK			DMC^c^	228.8	per case	0.5
					DMC IP	4035.8	per case	8.7
					DMC OP	17.4	per case	0
					DNMC	574.2	per case	1.2
					IC	258.5	per case	0.6
		US			DMC^c^	3691.0	per case	4.8
					DMC IP	5965.7	per case	7.8
					DMC OP	285.2	per case	0.4
					IC	1231.8	per case	1.6
		Canada			DMC^c^	261.6	per case	0.5
					DMC IP	2653.9	per case	4.8
					DMC OP	40.8	per case	0.1
					IC	1588.9	per case	2.9
		Canada			DMC^c^	899.3	per case	1.6
					DMC IP	8721.6	per case	15.7
					DMC OP	59.8	per case	0.1
					DMC NS	147.5	per case	0.3
					DNMC	373.8	per case	0.7
					IC	2691.2	per case	4.8
		Sweden			TC	1881.4	per case	3.3
		US and Canada			DMC IP	4459.2	per case	8.0 ^‡^
		UK			DMC IP	2052.1	per case	4.4
		Australia			DMC IP	8721.6	per case	13.4
4	Barnass et al. [39]	UK	1987	GBP				
					TC	3908.0	per case to the hospital	8.5
5	Choi et al. [40]	US	1987	USD				
					DMC IP	2174.5	per case to the nursing home	2.8
					DNMC	301.1		0.4
6	Hayes et al. [46]	UK	1989	GBP				
					DMC^c^	435.7	per case	0.9
					DMC IP	3061.0	per case	6.6
					DMC OP	196.7	per case	0.4
					DNMC	335.5	per case	0.7
					IC	1864.5	per case	4.0
7	Sockett and Roberts [52]	UK	1989	GBP				
					DMC^c^	2192.5	per case	4.8
					DMC IP	2523.5	per case	5.5
					DMC OP	257.0	per case	0.6
					DNMC	382.2	per case	0.8
					IC	1265.6	per case	2.7
8	Engvall et al. [29]	Sweden	1992	SEK				
					DMC^c^	6743.7	per case	12.0
					DMC IP	6280.6	per case	11.1
					DMC OP	463.1	per case	0.8
9	Dryden et al. [41]	UK	1993	GBP				
					DMC IP	2058.3	per case to the hospital	4.5
					DNMC	484.5		1.1
10	Roberts and Sockett [56]	UK	1992	GBP				
					TC	5379.9	per case	11.7
11	Gomez et al. [19]	US	1992	USD				
					TC	2551.1	per case	3.3
12	Spearing et al. [47]	Australia	1996	AUD				
					DMC IP	1774.2	per case to the hospital	2.7
					DNMC	998.8		1.5
					IC	403.6		0.6
13	Duff et al.* [58]	Canada	2001	CAD				
					DMC^r^	1571.4	per case	2.8
					IC	1715.6	per case	3.1
		UK		GBP				
					DMC^r^	2353.3	per case	5.1
					IC	3028.8	per case	6.6
		US		USD				
					DMC^r^	1945.6	per case	2.5
					IC	1947.2	per case	2.6
14	Roberts et al. [48]	UK	1995	USD				
					DMC OP	106.8	per case	0.2
15	Trevejo et al. [33]	US	1999	USD				
					DMC IP	13,706.7	per case	18.0
16	Adhikari et al. [33]	US	2001	USD				
					DMC^c^	2305.2	per case	3.0
					DMC IP	9530.6	per case	12.5
					DMC OP	476.5	per case	0.6
					IC	962.5	per case	1.3
17	Martin et al. [59]	Canada	2000	CAD				
					DMC IP	2481.7	per case	4.5
18	van den Brandhof et al. [57]	Netherlands	2002	EUR				
					TC	322.0	per case	0.6
19	Anil et al. [37]	Türkiye	2005	USD ^†^				
					DMC IP	21,179.8	per case	198.4
					DNMC	18,261.2	per case	171.1
20	Gil Prieto et al. [34]	Spain	2006	EUR				
					DMC IP	2702.3	per case	9.1
21	Broughton et al. [38]	Hong Kong SAR, China	2008	USD ^†^				
					DMC IP	2825.3	per case	5.8
22	Santos et al. [53]	UK	2008	GBP				
					DMC^c^	624.6	per case	1.4
					DMC IP	1098.1	per case	2.4
					DMC OP	151.2	per case	0.3
					DNMC	106.2	per case	0.2
					IC	527.9	per case	1.1
23	Herrick et al. [22]	US	1993	USD				
					DMC^c^	1368.8	per case	1.8
					DMC IP	2652.9	per case	3.5
					DMC OP	84.6	per case	0.1
24	Hoffmann et al. [54]	US	2009	USD				
					DMC^c^	8450.6	per case	11.1
					DMC IP	16,161.4	per case	21.2
					DMC OP	739.8	per case	1.0
25	Chen et al. [35]	Taiwan (Region of China)	2011	USD ^†^				
					DMC IP	545.9	per case	4.3 ^++^
26	Ailes et al. [49]	US	2008	USD				
					DMC^c^	874.4	per case	1.1
					DMC IP	1526.2	per case	2.0
					DMC OP	174.0	per case	0.2
					IC	1331.8	per case	1.7
27	Sundström et al. [55]	Sweden	2009	EUR				
					DMC^c^	2288.4	per case	4.1
					DMC IP	4306.0	per case	7.6
					DMC OP	270.8	per case	0.5
					DNMC	8.2	per case	0
28	Cummings et al. [42]	US	2011	USD				
					DMC IP	10,808.5	per case	14.2
29	Scharff et al. [50]	US	2011	USD				
					TC	2322.1	per case	3.0
30	Suijkerbuik et al. [51]	Netherlands	2012	EUR				
					DMC^c^	1357.5	per case	2.4
					DMC IP	2613.4	per case	4.6
					DMC OP	101.5	per case	0.2
					DNMC	4.3	per case	0
					IC	714.1	per case	1.3
31	Stephen and Barnett [60]	Australia	2013	AUD				
					DMC^c^	2862.8	per case	4.4
					DMC IP	5690.1	per case	8.7
					DMC OP	35.5	per case	0.1
32	Hoffmann et al. (Economic Research Service, Database) [32]	US	2018	USD				
					DMC^c^	3501.0	per case	4.6
					DMC IP	6812.2	per case	8.9
					DMC OP	189.8	per case	0.2
					IC	313.0	per case	0.4
33	Dmochowska et al. [61]	Poland	2018	EUR				
					DMC^c^	780.6	per case	4.2
					DMC IP	759.5	per case	4.1
					DMC OP	21.1	per case	0.1
34	Ford et al. [62]	Australia	2015	AUD				
					DMC^r^	205.3	per case	0.3
					IC	188.9	per case	0.3
35	Garrido-Estepa et al. [44]	Spain	2015	EUR				
					DMC IP	4759.4	per case	16.0
36	Lai et al. [5]	Taiwan (Region of China)	2015	TWD				
					DMC^r^	318.2	per case	2.5 ^++^
37	Collier et al. [43]	US	2014	USD				
					DMC IP	19,788.7	per use	25.9
38	Dhaliwal et al. [63]	US	2015	USD				
					DMC IP	9079.1	per case	11.9

DMC^r^ = a reported value retrieved from the literature as the mean of direct medical costs per case, which has been adjusted to USD in the year 2022 value. DMC^c^ = a calculated value as the mean of DMC per case (the total amount of DMC incurred divided by the total number of cases). DMCs IP = direct medical costs incurred from inpatient healthcare. DMCs OP = direct medical costs incurred from outpatient healthcare. DMCs NS (not specified) = costs are direct medical costs that are not categorized into direct medical costs for inpatient or outpatient healthcare services. DNMCs = direct non-medical costs. ICs = indirect costs. TCs = total costs. * Studies including cost data from more than two countries. ^†^ Costs using a different currency from one of the original countries (i.e., USD reported from Taiwan) were inflated using the original countries’ GDP deflator. **^§^** GDP per capita (current USD, 2022) from the World Bank [28]. ^‡^ Percentage of Canada’s GDP per capita (current USD) in 2022. ^++^ Percentage of China’s GDP per capita (current USD) in 2022.

**Table 4:** Healthcare costs by cost type related to invasive non-typhoidal *Salmonella* diseases (USD in 2022).

No.	Author	Country	Cost Year	Original Currency	Cost type	Cost in USD (2022)	Unit	% of GDP Per Capita ^§^
*15*	Trevejo et al. [33]	US	1999	USD				
					DMC IP	32,507.5	per case	42.6
*16*	Adhikari et al. [30]	US	2001	USD				
					DMC^c^	7003.8	per case	9.2
					DMC IP	25,838.4	per case	33.9
					DMC OP	469.4	per case	0.6
					IC	1768.8	per case	2.3
*20*	Gil Prieto et al. [34]	Spain	2006	EUR				
					DMC IP	5980.7	per case	20.2
*25*	Chen et al. [35]	Taiwan (Region of China)	2011	USD ^†^				
					DMC IP	1973.1	per case	15.5 ^++^

DMC^c^ = a calculated value as the mean of DMC per case (the total amount of DMC incurred divided by the total number of cases). DMCs IP = direct medical costs incurred from inpatient healthcare. DMCs OP = direct medical costs incurred from outpatient healthcare. ICs = indirect costs. ^†^ Costs using a different currency from one of the original countries (i.e., USD reported from Taiwan) were inflated using the original countries’ GDP deflator. **^§^** GDP per capita (current USD, 2022) from the World Bank [28]. ^++^ Percentage of China’s GDP per capita (current USD) in 2022.

**Table 5:** Number of outpatient visits and hospital bed-days by indication.

Indication	No.	Author	Number of Outpatient Visits	Hospital Bed-Days (Mean)	Country	Study Year
NTS	2	Curtin et al. [31]	-	10.6	Canada	1978
	3	Todd et al. [20]	-	12.3	UK	1983
	3	Todd et al. [20]	-	5.3	Canada	1977
	3	Todd et al. [20]	-	14	Canada	1978
	3	Todd et al. [20]	-	14	Australia	1974
	6	Hayes et al. [46]	1.6	11.1	UK	1989
	7	Sockett and Roberts [52]	-	6.4	UK	1988–1989
	8	Engvall et al. [29]	-	9.5	Sweden	1992
	10	Roberts and Sockett [56]	12 (median duration of illness)		UK	1988–1989
Invasive NTS	16	Adhikari et al. [30]	-	4.2	US	1993–2001
	17	Martin et al. [59]	-	4.1	Canada	1999–2000
	19	Anil et al. [37]	-	8.2	Türkiye	2005
	20	Gil Prieto et al. [34]	-	6.8	Spain	1997–2006
	23	Herrick et al. [22]	2 (median duration of illness)		US	2012
	25	Chen et al. [35]	-	5.7	Taiwan (Region of China)	2006–2008
	26	Ailes et al. [49]	4 (median duration of illness)		US	2008
	27	Sundström et al. [55]	-	5.1	Sweden	2010
	28	Cummings et al. [42]	-	5	US	2011
	30	Suijkerbuik et al. [51]	-	4	Netherlands	2012–2013
	31	Stephen and Barnett [60]	-	4.8	Australia	2013
	35	Garrido-Estepa et al. [44]	-	5	Spain	2010–2015
	38	Dhaliwal et al. [63]	-	5.6	US	2012–2015
	16	Adhikari et al. [30]	-	8.9	US	1993–2001
	20	Gil Prieto et al. [34]	-	17.3	Spain	1997–2006
	25	Chen et al. [35]	-	11.5	Taiwan (Region of China)	2006–2008

## Data Availability

All crude cost data are extracted from published articles and are publicly accessible. Access to the database searched and included in this review is available at https://www.ers.usda.gov/data-products/cost-estimates-of-foodborne-illnesses/ (accessed on 2 May 2024) (Economic Research Service, U.S. Department of Agriculture, Washington, D.C., United States of America).

## Supplementary Materials

The following supporting information can be downloaded at: https://www.mdpi.com/article/10.3390/vaccines12070758/s1, Table S1: PRISMA 2020 checklist; Table S2: Quality assessment; Table S3: Summary of the included articles.
