# Supplementary material for: The Economic Burden of Non-Typhoidal Salmonella and Invasive Non-Typhoidal Salmonella Infection: A Systematic Literature Review
Source: Vaccines (Basel). 2024 Jul 9;12(7):758. doi: 10.3390/vaccines12070758 (PMC11281589; doi:10.3390/vaccines12070758)
Supplement: Supplementary file 1 [file vaccines-12-00758-s001.zip › vaccines-3064156-supplementary.pdf]

Supplementary Materials; Table S1. PRISMA 2020 Checklist

Section and Topic	Item #	Checklist item	Location where item is reported
**TITLE**			
Title	1	Identify the report as a systematic review.	1
**ABSTRACT**			
Abstract	2	See the PRISMA 2020 for Abstracts checklist.	1
**INTRODUCTION**			
Rationale	3	Describe the rationale for the review in the context of existing knowledge.	1-2
Objectives	4	Provide an explicit statement of the objective(s) or question(s) the review addresses.	2
**METHODS**			
Eligibility criteria	5	Specify the inclusion and exclusion criteria for the review and how studies were grouped for the syntheses.	2, 4
Information sources	6	Specify all databases, registers, websites, organisations, reference lists and other sources searched or consulted to identify studies. Specify the date when each source was last searched or consulted.	2
Search strategy	7	Present the full search strategies for all databases, registers and websites, including any filters and limits used.	2-3
Selection process	8	Specify the methods used to decide whether a study met the inclusion criteria of the review, including how many reviewers screened each record and each report retrieved, whether they worked independently, and if applicable, details of automation tools used in the process.	2-4
Data collection process	9	Specify the methods used to collect data from reports, including how many reviewers collected data from each report, whether they worked independently, any processes for obtaining or confirming data from study investigators, and if applicable, details of automation tools used in the process.	2-4
Data items	10a	List and define all outcomes for which data were sought. Specify whether all results that were compatible with each outcome domain in each study were sought (e.g. for all measures, time points, analyses), and if not, the methods used to decide which results to collect.	4
	10b	List and define all other variables for which data were sought (e.g. participant and intervention characteristics, funding sources). Describe any assumptions made about any missing or unclear information.	4
Study risk of bias assessment	11	Specify the methods used to assess risk of bias in the included studies, including details of the tool(s) used, how many reviewers assessed each study and whether they worked independently, and if applicable, details of automation tools used in the process.	2-3
Effect measures	12	Specify for each outcome the effect measure(s) (e.g. risk ratio, mean difference) used in the synthesis or presentation of results.	4
Synthesis methods	13a	Describe the processes used to decide which studies were eligible for each synthesis (e.g. tabulating the study intervention characteristics and comparing against the planned groups for each synthesis (item #5)).	4
	13b	Describe any methods required to prepare the data for presentation or synthesis, such as handling of missing summary statistics, or data conversions.	4
	13c	Describe any methods used to tabulate or visually display results of individual studies and syntheses.	4
	13d	Describe any methods used to synthesize results and provide a rationale for the choice(s). If meta-analysis was performed, describe the model(s), method(s) to identify the presence and extent of statistical heterogeneity, and software package(s) used.	NA
	13e	Describe any methods used to explore possible causes of heterogeneity among study results (e.g. subgroup analysis, meta-regression).	NA
	13f	Describe any sensitivity analyses conducted to assess robustness of the synthesized results.	NA
Reporting bias assessment	14	Describe any methods used to assess risk of bias due to missing results in a synthesis (arising from reporting biases).	NA
Certainty assessment	15	Describe any methods used to assess certainty (or confidence) in the body of evidence for an outcome.	NA

Section and Topic	Item #	Checklist item	Location where item is reported
**RESULTS**			
Study selection	16a	Describe the results of the search and selection process, from the number of records identified in the search to the number of studies included in the review, ideally using a flow diagram.	4-5
	16b	Cite studies that might appear to meet the inclusion criteria, but which were excluded, and explain why they were excluded.	-
Study characteristics	17	Cite each included study and present its characteristics.	5-15
Risk of bias in studies	18	Present assessments of risk of bias for each included study.	15, Supplementary Materials; Table S2
Results of individual studies	19	For all outcomes, present, for each study: (a) summary statistics for each group (where appropriate) and (b) an effect estimate and its precision (e.g. confidence/credible interval), ideally using structured tables or plots.	5-16
Results of syntheses	20a	For each synthesis, briefly summarise the characteristics and risk of bias among contributing studies.	NA
	20b	Present results of all statistical syntheses conducted. If meta-analysis was done, present for each the summary estimate and its precision (e.g. confidence/credible interval) and measures of statistical heterogeneity. If comparing groups, describe the direction of the effect.	NA
	20c	Present results of all investigations of possible causes of heterogeneity among study results.	NA
	20d	Present results of all sensitivity analyses conducted to assess the robustness of the synthesized results.	NA
Reporting biases	21	Present assessments of risk of bias due to missing results (arising from reporting biases) for each synthesis assessed.	NA
Certainty of evidence	22	Present assessments of certainty (or confidence) in the body of evidence for each outcome assessed.	NA
**DISCUSSION**			
Discussion	23a	Provide a general interpretation of the results in the context of other evidence.	16
	23b	Discuss any limitations of the evidence included in the review.	16
	23c	Discuss any limitations of the review processes used.	16
	23d	Discuss implications of the results for practice, policy, and future research.	16-17
**OTHER INFORMATION**			
Registration and protocol	24a	Provide registration information for the review, including register name and registration number, or state that the review was not registered.	-
	24b	Indicate where the review protocol can be accessed, or state that a protocol was not prepared.	-
	24c	Describe and explain any amendments to information provided at registration or in the protocol.	-
Support	25	Describe sources of financial or non-financial support for the review, and the role of the funders or sponsors in the review.	17
Competing interests	26	Declare any competing interests of review authors.	17
Availability of data, code and other materials	27	Report which of the following are publicly available and where they can be found: template data collection forms; data extracted from included studies; data used for all analyses; analytic code; any other materials used in the review.	17

From: Page MJ, McKenzie JE, Bossuyt PM, Boutron I, Hoffmann TC, Mulrow CD, et al. The PRISMA 2020 statement: an updated guideline for reporting systematic reviews. BMJ 2021;372:n71. doi: 10.1136/bmj.n71

For more information, visit: http://www.prisma-statement.org/

Supplementary Materials; Table S2. Quality assessment

Study ID	Authors	A) Analytical framework: what costs should have been measured?						B) Methodology and data: how well were resource use and productivity losses measured?									
		1. Was the motivation and perspective of the study stated?	2. Was the appropriate epidemiologic approach taken?	3. Was the study question well specified?				4. Was an appropriate method(s) of quantification used, such that					5. Was the resource quantification method(s) well executed?			6. Were healthcare resources valued appropriately?	7. Was the approach for valuing production losses justified, and assumptions valid?
				3.1 Were all relevant, non-trivial cost components and their stakeholders identified?	3.2 Were necessary timeframes specified?	3.3 Was a case of disease or risk factor adequately and appropriately defined?	3.4 Was the counterfactual population occurrence plausible and meaningful?	4.1 additional, or excess, costs were measured?	4.2 only costs specific to (caused by) the health problem were included (confounders controlled)?	4.3 all important effects were captured?	4.4 important differences across subpopulations were accounted for?	4.5 the required level of detail could be provided?	5.1 For population-based studies, were cost allocation methods, data and assumptions valid?	5.2 For person-based studies, were appropriate statistical tests performed and reported?	5.3 Were data representative of the study population?		
1	Cohen et al.	Y	Y	Y	Y	Y	NA	N	Y	Y	Y	Y	NA	Y	Y	Y	Y
2	Curtin et al.	Y	Y	Y	Y	Y	NA	N	Y	Y	Y	Y	NA	Y	Y	Y	Y
3	Todd et al.	Y	Y	Y	Y	Y	NA	N	Y	Y	Y	Y	NA	Y	Y	Y	Y
4	Barnass et al.	Y	Y	Y	Y	Y	NA	N	Y	Y	NA	N	NA	Y	Y	Y	NA
5	Choi et al.	Y	Y	N	Y	Y	NA	N	N	N	NA	N	Y	NA	Y	Y	NA
6	Hayes et al.	Y	Y	Y	Y	Y	NA	N	Y	Y	Y	Y	Y	NA	Y	Y	Y
7	Sockett & Roberts	Y	Y	Y	Y	Y	NA	Y	Y	Y	Y	Y	Y	NA	Y	Y	Y
8	Engvall et al.	Y	Y	N	Y	Y	NA	Y	Y	Y	Y	Y	Y	NA	Y	Y	NA
9	Dryden et al.	Y	Y	Y	Y	Y	Y	Y	Y	Y	NA	Y	NA	Y	Y	Y	NA
10	Roberts & Sockett	Y	Y	Y	Y	Y	NA	Y	Y	Y	Y	Y	Y	NA	Y	Y	NA
11	Gomez et al.	Y	Y	N	Y	Y	NA	N	N	Y	N	Y	Y	NA	N	Y	NA
12	Spearing et al.	Y	Y	Y	Y	Y	NA	N	Y	Y	Y	Y	NA	Y	Y	Y	Y
13	Duff et al.	Y	Y	N	Y	Y	NA	Y	Y	Y	Y	Y	Y	NA	Y	Y	Y
14	Roberts et al.	Y	Y	N	Y	Y	Y	Y	Y	Y	Y	Y	Y	NA	Y	Y	Y
15	Trevejo et al.	Y	Y	Y	Y	Y	NA	N	N	Y	Y	Y	NA	Y	Y	Y	NA
16	Adhikari et al.	Y	Y	Y	Y	Y	NA	N	Y	Y	Y	Y	Y	NA	Y	Y	Y
17	Martin et al.	Y	Y	N	Y	Y	NA	N	Y	Y	Y	Y	NA	Y	Y	Y	NA
18	van den Brandhof et al.	Y	Y	Y	Y	Y	NA	NA	Y	Y	Y	Y	Y	NA	Y	Y	NA
19	Anil et al.	Y	Y	Y	Y	Y	NA	Y	Y	Y	Y	Y	NA	Y	Y	Y	NA
20	Gil Prieto et al.	Y	Y	Y	Y	Y	NA	NA	Y	Y	Y	Y	Y	NA	Y	Y	NA
21	Broughton et al.	Y	Y	Y	Y	Y	NA	NA	Y	Y	Y	Y	Y	NA	Y	Y	NA
22	Santos et al.	Y	Y	Y	Y	Y	NA	NA	Y	Y	Y	Y	NA	Y	Y	Y	Y
23	Herrick et al.	Y	Y	Y	Y	Y	NA	NA	Y	Y	Y	Y	Y	NA	Y	Y	Y
24	Hoffmann et al.	Y	Y	Y	Y	Y	Y	Y	Y	Y	Y	Y	Y	NA	Y	Y	Y
25	Chen et al.	Y	Y	Y	Y	Y	Y	Y	Y	Y	Y	Y	Y	NA	Y	Y	NA
26	Ailes et al.	Y	Y	Y	Y	Y	NA	NA	Y	Y	Y	Y	NA	Y	Y	Y	Y
27	Sundström et al.	Y	Y	Y	Y	Y	Y	Y	Y	Y	Y	Y	Y	NA	Y	Y	NA
28	Cummings et al.	Y	Y	Y	Y	Y	NA	NA	Y	Y	Y	Y	NA	Y	Y	Y	NA
29	Scharff et al.	Y	Y	Y	Y	Y	NA	NA	Y	Y	Y	Y	Y	NA	Y	Y	Y

30	Suijkerbuik et al.	Y	Y	Y	Y	Y	NA	NA	Y	Y	Y	Y	NA	Y	Y	Y	Y
31	Stephen & Barnett	Y	Y	Y	Y	Y	Y	Y	Y	Y	Y	Y	Y	NA	Y	Y	NA
32	Hoffmann et al. (Economic Research Service)	NA	NA	NA	NA	NA	NA	NA	NA	NA	NA	NA	NA	NA	NA	NA	NA
33	Dmochowska et al.	Y	Y	Y	Y	Y	NA	NA	Y	Y	Y	Y	Y	NA	Y	Y	NA
34	Ford et al.	Y	Y	Y	Y	Y	NA	NA	Y	Y	Y	Y	Y	NA	Y	Y	Y
35	Garrido-Estepa et al.	Y	Y	Y	Y	Y	NA	NA	Y	Y	Y	Y	Y	NA	Y	Y	NA
36	Lai et al.	Y	Y	Y	Y	Y	NA	NA	Y	Y	Y	Y	Y	NA	Y	NA	Y
37	Collier et al.	Y	Y	Y	Y	Y	NA	NA	Y	Y	Y	Y	Y	NA	Y	Y	NA
38	Dhaliwal et al.	Y	Y	Y	Y	Y	NA	NA	Y	Y	Y	Y	Y	NA	Y	Y	NA

Supplementary Materials; Table S2. Quality assessment (Cont.)

Study ID	Authors	C) Analysis and reporting								Score (0 - 25)					Quality	
		8. Did the analysis address the study question?	9. Was a range of estimates presented?	10. Were the main uncertainties identified?	11. Was a sensitivity analysis performed	12. Was adequate documentation and justification given for cost components, data and sources, assumptions and methods?	13. Was uncertainty around the estimates and its implications adequately discussed?	14. Were important limitations discussed regarding the cost components, data, assumptions and methods?	15. Were the results presented at the appropriate level of detail to answer the study question (cost components; disease subtypes, severity, stage; subpopulation groups, cost bearers)?	Y	N	NA	NA without double (for population, person)	Y + NA	%	Low: <70, Medium: 70-85, High: >85)
1	Cohen et al.	Y	N	N	N	Y	N	N	Y	16	6	2	1	17	68%	Low
2	Curtin et al.	Y	N	Y	N	Y	Y	Y	Y	19	3	2	1	20	80%	Medium
3	Todd et al.	Y	N	N	N	Y	N	N	N	15	7	2	1	16	64%	Low
4	Barnass et al.	Y	N	N	N	N	N	N	N	11	9	4	3	14	56%	Low
5	Choi et al.	Y	N	N	N	Y	N	N	Y	10	10	4	3	13	52%	Low
6	Hayes et al.	Y	N	Y	N	Y	Y	Y	Y	19	3	2	1	20	80%	Medium
7	Sockett & Roberts	Y	N	Y	N	Y	Y	Y	Y	20	2	2	1	21	84%	Medium
8	Engvall et al.	Y	N	Y	N	Y	Y	Y	Y	18	3	3	2	20	80%	Medium
9	Dryden et al.	Y	N	Y	N	Y	Y	Y	Y	19	2	3	2	21	84%	Medium
10	Roberts & Sockett	Y	N	Y	N	Y	Y	Y	Y	19	2	3	2	21	84%	Medium
11	Gomez et al.	Y	Y	Y	N	Y	Y	N	Y	14	7	3	2	16	64%	Low
12	Spearing et al.	Y	N	Y	N	Y	Y	N	Y	18	4	2	1	19	76%	Medium
13	Duff et al.	Y	Y	Y	Y	Y	Y	Y	Y	21	1	2	1	22	88%	High
14	Roberts et al.	Y	Y	Y	Y	Y	Y	N	Y	21	2	1	0	21	84%	Medium
15	Trevejo et al.	Y	Y	Y	N	Y	Y	Y	Y	18	3	3	2	20	80%	Medium
16	Adhikari et al.	Y	Y	Y	Y	Y	Y	Y	Y	21	1	2	1	22	88%	High
17	Martin et al.	Y	N	N	N	Y	Y	Y	Y	16	5	3	2	18	72%	Medium

18	van den Brandhof et al.	Y	Y	Y	Y	Y	Y	Y	Y	20	0	4	3	23	92%	High
19	Anil et al.	Y	Y	Y	N	Y	Y	Y	Y	20	1	3	2	22	88%	High
20	Gil Prieto et al.	Y	N	Y	N	Y	Y	Y	Y	18	2	4	3	21	84%	Medium
21	Broughton et al.	Y	Y	Y	N	Y	Y	Y	Y	19	1	4	3	22	88%	High
22	Santos et al.	Y	N	Y	Y	Y	Y	Y	Y	20	1	3	2	22	88%	High
23	Herrick et al.	Y	Y	Y	Y	Y	Y	Y	Y	21	0	3	2	23	92%	High
24	Hoffmann et al.	Y	Y	Y	Y	Y	Y	Y	Y	23	0	1	0	23	92%	High
25	Chen et al.	Y	Y	Y	N	Y	Y	Y	Y	21	1	2	1	22	88%	High
26	Ailes et al.	Y	Y	Y	Y	Y	Y	Y	Y	21	0	3	2	23	92%	High
27	Sundström et al.	Y	Y	Y	Y	Y	Y	Y	Y	22	0	2	1	23	92%	High
28	Cummings et al.	Y	Y	Y	N	Y	Y	Y	Y	19	1	4	3	22	88%	High
29	Scharff et al.	Y	Y	Y	Y	Y	Y	Y	Y	21	0	3	2	23	92%	High
30	Suijkerbuik et al.	Y	N	Y	N	Y	Y	Y	Y	19	2	3	2	21	84%	Medium
31	Stephen & Barnett	Y	Y	Y	Y	Y	Y	Y	Y	22	0	2	1	23	92%	High
32	Hoffmann et al. (Economic Research Service)	NA	NA	NA	NA	NA	NA	NA	NA							Database
33	Dmochowska et al.	Y	Y	Y	N	Y	Y	Y	Y	19	1	4	3	22	88%	High
34	Ford et al.	Y	Y	Y	Y	Y	Y	Y	Y	21	0	3	2	23	92%	High
35	Garrido-Estepa et al.	Y	Y	Y	N	Y	Y	Y	Y	19	1	4	3	22	88%	High
36	Lai et al.	Y	Y	Y	Y	Y	Y	Y	Y	20	0	4	3	23	92%	High
37	Collier et al.	Y	Y	Y	Y	Y	Y	Y	Y	20	0	4	3	23	92%	High
38	Dhaliwal et al.	Y	Y	Y	N	Y	Y	Y	Y	19	1	4	3	22	88%	High

Adapted from: Larg, A. and J. Moss, *Cost-of-Illness Studies: A Guide to Critical Evaluation.* PharmacoEconomics, 2011. **29**: p. 653-71.

Supplementary Materials; Table S3. Summary of the included articles.

No	Author	Publication year	Country	Region	Income group	Indication	Cost type	Cost perspective	Setting	Cost source
1	Cohen *et al.*	1978	United States	Americas	HIC	NTS	DMC (IP & OP) DNMC IC	Societal	Community outbreak	**Questionnaire**

2	Curtin *et al.*	1984	Canada	Americas	HIC	NTS	DMC (IP & OP)<br>DNMC<br>IC	Societal	Community	**National statistics**
3	Todd *et al.*	1985	Australia, Canada, Sweden, United Kingdom, United States	Multiple regions	HIC	NTS	DMC (IP, OP & NS)<br>DNMC<br>IC	Societal	Community outbreaks	**Other studies and national reports**
4	Barnass *et al.*	1989	United Kingdom	Europe	HIC	NTS	TC	Healthcare system	Nosocomial outbreak	**Hospital data**
5	Choi *et al.*	1990	United States	Americas	HIC	NTS	DMC (IP)<br>DNMC	Healthcare system	Nosocomial outbreak	**Investigation**
6	Hayes *et al.*	1991	United Kingdom	Europe	HIC	NTS	DMC (IP & OP)<br>DNMC<br>IC	Societal	Community outbreak	**Questionnaire**
7	Sockett & Roberts	1991	United Kingdom	Europe	HIC	NTS	DMC (IP & OP)<br>DNMC<br>IC	Societal	Community	**National survey**
8	Engvall *et al.*	1992	Sweden	Europe	HIC	NTS	DMC (IP & OP)	Patient	Community	**National statistics**

9	Dryden *et al.*	1994	United Kingdom	Europe	HIC	NTS	DMC (IP) DNMC	Healthcare system	Nosocomial outbreak	**Investigation**
10	Roberts & Sockett	1994	United Kingdom	Europe	HIC	NTS	TC	Societal	Community	**National survey and outbreak studies**
11	Gomez *et al.*	1997	United Kingdom, United States	Europe, Americas	HIC	NTS	TC	Societal	Community	**Other studies**
12	Spearing *et al.*	2000	Australia	Western Pacific	HIC	NTS	DMC (IP) DNMC IC	Societal	Nosocomial outbreak	**Hospital data**
13	Duff *et al.*	2003	Canada, United Kingdom, United States	Americas, Europe	HIC	NTS	DMC (IP & OP combined) IC	Societal	Community	**Other studies and expert opinion**
14	Roberts *et al.*	2003	United Kingdom	Europe	HIC	NTS	DMC (OP)	Societal	Community	**Questionnaire and national data**
15	Trevejo *et al.*	2003	United States	Americas	HIC	NTS and iNTS	DMC (IP)	Patient	Community	**Hospital data**

16	Adhikari *et al.*	2004	United States	Americas	HIC	NTS and iNTS	DMC (IP & OP)<br>IC	Societal	Community	**National claims data**
17	Martin *et al.*	2004	Canada	Americas	HIC	NTS	DMC (IP)	Societal	Community	**Questionnaire and national data**
18	Van den Brandhof *et* [illegible]	2004	Netherlands	Europe	HIC	NTS	TC	Societal	Community	**Community-based cohort study**
19	Anil *et al.*	2009	Turkey	Europe	UMIC	NTS	DMC (IP)<br>DNMC	Healthcare system	Nosocomial outbreak	**Investigation**
20	Gil Prieto *et al.*	2009	Spain	Europe	HIC	NTS and iNTS	DMC (IP)	Patient	Community	**Hospital data**
21	Broughton *et al.*	2010	Hong Kong	Western Pacific	HIC	NTS	DMC (IP)	Patient	Community	**Hospital data**
22	Santos *et al.*	2010	United Kingdom	Europe	HIC	NTS	DMC (IP & OP)<br>DNMC<br>IC	Societal	Community	**Questionnaire**
23	Herrick *et al.*	2012	United States	Americas	HIC	NTS	DMC (IP & OP)	Societal	Community outbreak	**Other studies and national data**

24	Hoffmann *et al.*	2012	United States	Americas	HIC	NTS	DMC (IP, OP & NS)	Societal	Community	**Surveillance data**
25	Chen *et al.*	2012	Taiwan (Region of China)	Western Pacific	HIC	NTS and iNTS	DMC (IP)	Healthcare system	Community	**National claims data**
26	Ailes *et al.*	2013	United States	Americas	HIC	NTS	DMC (IP & OP)<br>DNMC<br>IC	Societal	Community outbreak	**Community survey**
27	Sundström *et al.*	2014	Sweden	Europe	HIC	NTS	DMC (IP & OP)<br>DNMC	Societal	Community	**National data**
28	Cummings *et al.*	2016	United States	Americas	HIC	NTS	DMC (IP)	Healthcare system	Community	**National survey**
29	Scharff *et al.*	2016	United States	Americas	HIC	NTS	TC	Societal	Community outbreaks	**Surveillance data**
30	Suijkerbuik *et al.*	2016	Netherlands	Europe	HIC	NTS	DMC (IP & OP)<br>DNMC<br>IC	Societal	Community outbreak	**Case-control study and national data**
31	Stephen & Barnett	2017	Australia	Western Pacific	HIC	NTS	DMC (IP & OP)	Societal	Community	**National data and other study**

32	Hoffmann *et al.* [illegible]	2018	United States	Americas	HIC	NTS	DMC (IP & OP)<br>IC	Societal	Community	**Surveillance data and other studies**
33	Dmochowska *et al.*	2019	Poland	Europe	HIC	NTS	DMC (IP & OP)	Societal	Community	**National data**
34	Ford *et al.*	2019	Australia	Western Pacific	HIC	NTS	DMC (IP & OP combined)<br>IC	Societal	Community	**National survey**
35	Garrido-Estepa *et al.*	2019	Spain	Europe	HIC	NTS	DMC (IP)	Patient	Community	**National data**
36	Lai *et al.*	2020	Taiwan (Region of China)	Western Pacific	HIC	NTS	DMC (IP & OP combined)	Healthcare system	Community	**National claims data**
37	Collier *et al.*	2021	United States	Americas	HIC	NTS	DMC (IP)	Healthcare system	Community	**National claims data**
38	Dhaliwal *et al.*	2021	United States	Americas	HIC	NTS	DMC (IP)	Societal	Community	**National data**

HIC = High-Income Countries. UMIC = Upper Middle-Income Countries. NTS = Non-typhoidal Salmonella disease. DMC = Direct Medical Costs. IP = Inpatient. OP = Outpatient. DMC NS (Not specified) costs are direct medical costs that are not categorized into direct medical costs for inpatient nor direct medical costs for outpatient healthcare services. DNMC = Direct Non-Medical Costs. IC = Indirect Costs. TC = Total Costs. The full bibliography is in the main text.
